# The relationship between vertebrobasilar artery calcification and intracranial atherosclerosis-related occlusion in thrombectomy

**DOI:** 10.3389/fneur.2022.965362

**Published:** 2022-10-04

**Authors:** Nannan Han, Gejuan Zhang, Shiyao Yang, Haojun Ma, Hanming Ge, Xiao Zhang, Shilin Li, Yanfei Wang, Xiaonan Fan, Yanling Yin, Yanjun Gao, Wenzhen Shi, Xiaobo Zhang, Mingze Chang, Ye Tian

**Affiliations:** ^1^Department of Neurology, Xi'an No. 3 Hospital, The Affiliated Hospital of Northwest University, Xi'an, China; ^2^Xi'an Key Laboratory of Cardiovascular and Cerebrovascular Diseases, Xi'an No. 3 Hospital, The Affiliated Hospital of Northwest University, Xi'an, China; ^3^The College of Life Sciences, Northwest University, Xi'an, China; ^4^Medical Research Center, Xi'an No. 3 Hospital, The Affiliated Hospital of Northwest University, Xi'an, China; ^5^Department of Radiology, Xi'an No. 3 Hospital, The Affiliated Hospital of Northwest University, Xi'an, China

**Keywords:** acute ischemic stroke, thrombectomy, large vessel occlusion, vertebrobasilar artery calcification, intracranial atherosclerosis-related occlusion

## Abstract

**Background and purpose:**

Distinguishing between intracranial atherosclerosis-related occlusion (ICAS-O) and non-ICAS-O can benefit strategies of identifying the need for surgical plans prior to thrombectomy. We investigated the association between vertebrobasilar artery calcification (VBAC) and ICAS-O in acute ischemic stroke patients undergoing thrombectomy.

**Methods:**

Patients were recruited from a prospective single-center registration study who had undergone thrombectomy between October 2017 and October 2021. The enrolled patients were divided into ICAS-O and non-ICAS-O, as determined by the intraarterial therapy process. The occurrences of VBAC were recorded on intracranial non-contrast computed tomography (NCCT) scans before thrombectomy. The association between VBAC and ICAS-O was assessed using binary logistic regression.

**Results:**

A total of 2732 patients who had undergone digital subtraction angiography were reviewed, and 314 thrombectomy patients (mean age: 65.4 years, 36.6% female) with NCCT were enrolled in this study. VBAC was detected before thrombectomy in 113 (36%) out of 314 patients. Age, hypertension, and diabetes were associated with VBAC, and a higher frequency of VBAC was identified in patients presenting posterior circulation. ICAS-O accounts for 43% (135/314) in eligible patients. From multivariable analyses, VBAC was identified as an independent predictor of ICAS-O (adjusted odds ratio, 6.16 [95% CI, 2.673–14.217], *P* < 0.001). Meanwhile, the (VBAC[+] atrial fibrillation[-]) group displayed higher rates of ICAS-O than the (VBAC[-] atrial fibrillation [-]) group (*P* < 0.001).

**Conclusions:**

We demonstrated that VBAC is an independent risk factor for ICAS-O in patients who underwent thrombectomy. Patients free of atrial fibrillation with VBAC have more trend to be ICAS-O.

## Introduction

Thrombectomy is a standard therapy used for acute ischemic stroke (AIS) with large vessel occlusion (LVO) (1). The etiology of AIS caused by LVO is predominantly of the cardioembolic subtype (2). However, LVO based on intracranial atherosclerosis is a common cause of AIS in Asia (3–5) and can affect individuals of any ethnicity (6).

The technical requirements for intraarterial therapy of atherosclerosis-related occlusion (ICAS-O) are higher than that of embolic occlusion (7). In the process of ICAS-O endovascular therapy, about 1/3 of patients will experience intraoperative reocclusion (8, b) requiring rescue treatments, including balloon angioplasty, stenting, and intra-arterial glycoprotein IIb/IIIa inhibitor (GPI) infusion (4, 8–11). Moreover, administering GPI intravenously prevents reocclusion caused by vascular endothelial injury and platelet aggregation after balloon angioplasty or emergency stent implantation (4, 12). Therefore, it is important and of value to confirm ICAS-O before thrombectomy.

At present, there is no imaging method to quickly determine the type of occlusion (8, b). Vertebrobasilar artery calcification (VBAC) on non-contrast computed tomography (NCCT) is widely used to visualize and represent intracranial atherosclerosis (13, 14). Therefore, this study was designed to investigate the relationship between VBAC and ICAS-O in order to potentially quickly determine the types of occlusions.

## Methods

### Patients and population

The patients in this study came from a registered study (A New Parameter Derived From DSA to Evaluate Cerebral Perfusion, NCT03607565) that continuously collects cases in the Department of Neurology of the Affiliated Hospital of Northwest University, with cases reviewed between October 2017 and October 2021. The inclusion criteria of this study were patients with: (1) Acute ischemic stroke caused by large artery occlusion (ICA, MCA M1-M2 segment, ACA, VA, BA); (2) Previous endovascular interventional therapy (thrombus aspiration, stent retrievers thrombectomy, emergency balloon angiography, and emergency stent implantation); (3) Intracranial NCCT includes the initial segment of V4 at foramen magnum before thrombectomy or follow-up after 24h. The exclusion criteria were patients with (1) Cerebral infarction caused by blockage of the venous system; (2) Incomplete head CT.

The local Institutional Review Board approved this study at the Affiliated Hospital of Northwest University (No. SYXSLL-2018-010), and the requirement for informed patient consent was waived due to the retrospective nature of this study.

### Clinical assessment

We used the following baseline characteristics of patients from the utilized database: age, gender, previous history of stroke, hypertension, diabetes, atrial fibrillation (AF) (AF was determined by electrocardiogram or medical history at emergency department, rather than developing during hospitalization), current smoking, initial stroke severity assessed by the National Institutes of Health Stroke Scale (NIHSS), and the location of the blocked artery.

### Radiological assessment

Intracranial NCCT was performed by two items of equipment (SOMATOM Definition Flash SIEMENS and Optima CT 680 GE) randomly and automatically uploaded to the picture archiving and communication systems (PACS) with a designated layer thickness of 5mm. If the V4 segment of the vertebral artery is not fully included, or motor artifacts appear in the NCCT before thrombectomy, then a follow-up NCCT concluded the foramen magnum (the V4 segment of the vertebral artery can be fully contained) as an alternative. A 24h follow-up CT after thrombectomy was performed to prevent artery enhancement after internventional therapy (15). Following this step, reader 1 (N.H.) reviewed the head CT from PACS and searched the high-density signal along the vessel from the V4 segment to the top of the basilar artery layer by layer. The number of pixels in the area above 130 (16) Hounsfield units (HU) was recorded as VBAC(+). Otherwise, all other values were recorded as VBAC(-). At last, reader 1 download the NCCT form PACS in dicom format and sends it to reader 2 (H.M. binded to clinical data) for re-evaluation.

We determined the ICAS-O during the intraarterial therapy process when there was (1) residual stenosis of 50% or more after initial thrombectomy or (2) intraprocedural restenosis or reocclusion or (3) evidence of hypoperfusion in territories downstream of the stenosis, and (4) other differential diagnoses such as vasospasm or vessel dissection have been ruled out. This is typically established by repeating the angiogram 10 min after a successful thrombectomy attempt (8, 9). The dissections were determined by an intimal flap proximal to occlusion or definitive double lumen and intimal flap on cerebral angiography after initial aspiration thrombectomy confirmed by neurological surgeon with more than 13 years of experience.

All NCCT data were obtained and viewed in PACS by neuroradiologists with more than 5 years of experience. In addition, the ICAS-O was confirmed by neurological surgeons with more than 13 years of experience. Similar to the evaluation of the other neuroimages, all images were analyzed separately by a neurologist and a neuroradiologist. All disagreements were resolved by reaching a consensus. If no consensus could be reached, another reviewer made the final decision.

### Statistical analysis

All enrolled patients were divided into VBAC(+) and VBAC(-) groups identified via NCCT. Continuous variables were expressed as the mean ± standard deviation or median (inter-quartile range, IQR), and the student's *t*-test was used to detect differences between groups. For categorical variables, frequency and percentage were used to summarize data, and between-group comparisons were performed via the Chi-square, Continuity Correction, or Fisher's exact test, as appropriate.

Multivariable logistic regression analysis was performed to identify the association between ICAS-O and non-ICAS-O. Variables with *P* < 0.1 in the univariate analysis were included in the multivariate logistic regression analysis using the ENTER method. Additionally, the percentage between-group comparisons in VBAC and AF groups were performed via the Chi-square or Continuity Correction. All data were analyzed using SPSS 22.0 (IBM, Armonk, NY, USA) with a significance level of *p* < 0.05 (2-sided).

## Results

**Figure 1:**
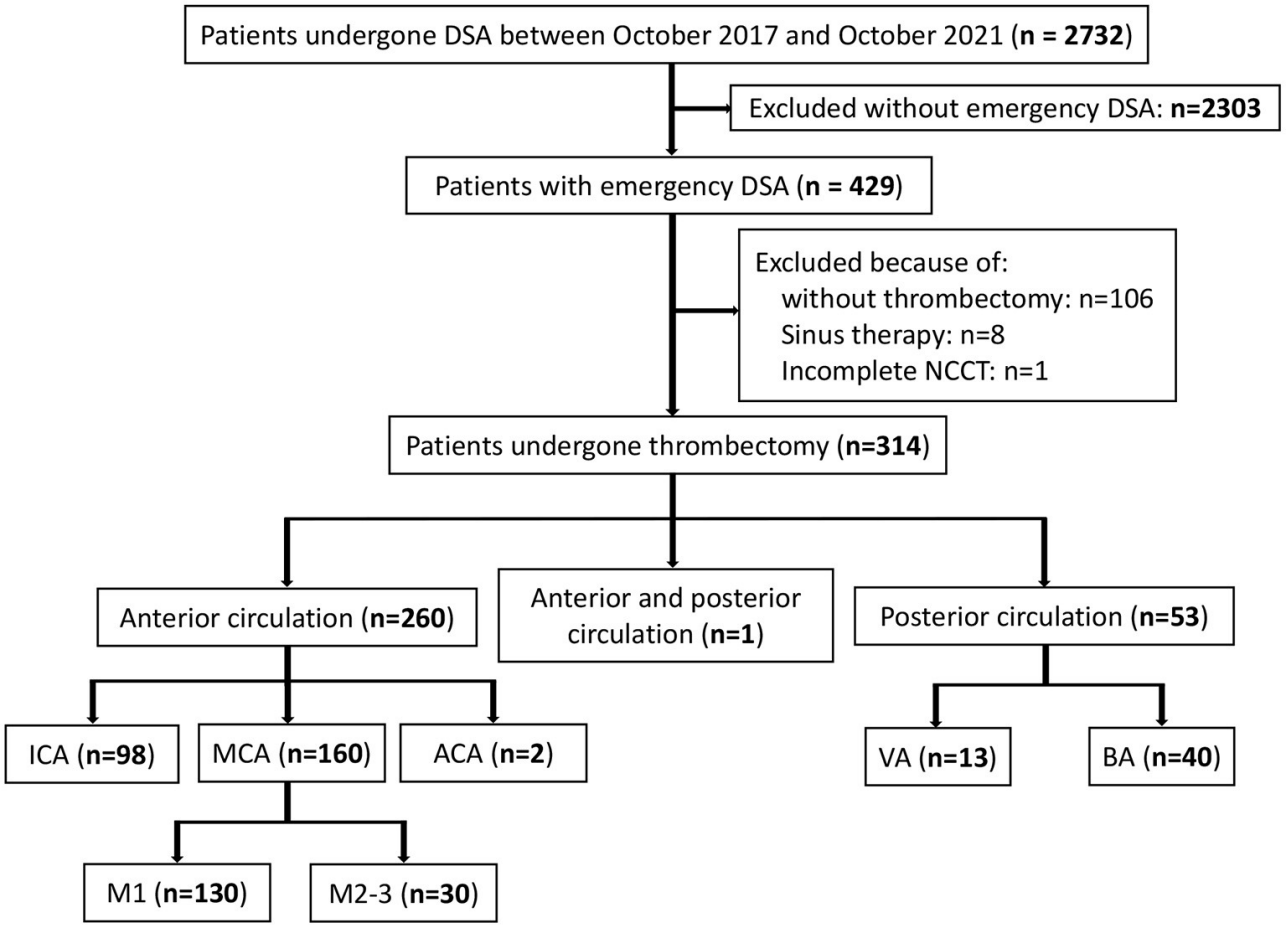
Patients flow-chart of the cohort. DSA indicates digital subtraction angiography; NCCT, non-contrast CT; ICA, internal carotid artery MCA, middle cerebral artery; VA, vertical artery and BA, basilar artery.

Our retrospective analysis identified 2,732 eligible patients in the database between October 2017 and October 2021. Among the 429 patients with emergency digital subtraction angiography (DSA), 314 met the study-specific inclusion criteria and were enrolled in this study. A total of 115 patients were excluded resulting from 106 patients not meeting the criteria for thrombectomy or the guardian refusing to perform thrombectomy, 8 patients having undergone sinus thrombectomy, and 1 patient having an incomplete NCCT (Figure 1).

Of the 314 patients in the study, the mean age was 65.4 ± 12.8 years, and 36.6% of this population were female. The Kappa = 0.993, *P* < 0.001 in two readers for VBAC. A summary of baseline characteristics of the entire patient population is shown in Table 1. In 36% (113/314) of enrolled cases, VBAC was present. The probability of VBAC was 30.8% (80/260) in patients with the anterior circulation occlusion and 62.3% (33/53) in the posterior circulation occlusion. Patients with VBAC present were older (*P* = 0.02), had higher rates of hypertension (*P* < 0.001), diabetes (*P* = 0.031), and VBAC were more likely to present in the posterior circulation (*P* < 0.001).

**Table 1:** Baseline characteristics.

**Characteristics**	**VBAC** **(*n* = 113)**	**Non-VBAC** **(*n* = 201)**	***p*-value**
Age, mean (SD)	67.5 (11.1)	64.2 (13.6)	0.020*
Gender female, *n* (%)	36 (31.9)	79 (39.3)	0.189
Previous ischemic stroke, *n* (%)	26 (23.0)	36 (17.9)	0.276
Hypertension, *n* (%)	84 (74.3)	103 (51.2)	<0.001*
Diabetes, *n* (%)	30 (26.5)	33 (16.4)	0.031*
Atrial fibrillation, *n* (%)	40 (35.4)	68 (33.8)	0.779
Current smoking, *n* (%)	42 (37.2)	64 (31.8)	0.338
Baseline NIHSS, median (IQR)	14 (8, 19)	13 (7.5, 18)	0.440
Posterior circulation	33 (29.2)	20 (10.0)	<0.001*

VBAC, vertebrobasilar artery calcification; SD, standard deviation; NIHSS, national institute of health stroke scale; IQR, interquartile range. *p < 0.05 indicates statistical significance.

The baseline characteristics (Table 2) of the ICAS-O group were similar to those of the non-ICAS-O group, except for age (61.5 ± 10.9 vs. 68.3 ± 13.5, respectively; *P* < 0.001), gender (female, 20 vs. 49.2%, respectively; *P* < 0.001), hypertension (69.6 vs. 52%, respectively; *P* = 0.001), diabetes (27.4 vs. 14.5%, respectively; *P* = 0.004), AF (3 vs. 58.1%, respectively; *P* < 0.001), current smoking (45.9% vs. 24.6%, respectively; *P* < 0.001), baseline NIHSS (9 [5, 16] vs. 15 [11, 19], respectively; *P* < 0.001), posterior circulation (30.4% vs. 6.7%, respectively; *P* < 0.001), and VBAC (50.4 vs. 25.1%, respectively; *P* < 0.001). In the multivariable analyses, the independent predictor of ICAS-O were female (adjusted odds ratio [aOR], 0.349 [95% CI, 0.155–0.787], *P* = 0.011), AF (aOR, 0.01 [95% CI, 0.003–0.046], *P* < 0.001), baseline NIHSS (aOR, 0.93 [95%CI 0.887-0.973], *P* = 0.002), posterior circulation (aOR, 4.04 [95% CI, 1.454–11.242], *P* < 0.007), and VBAC (aOR, 6.16 [95% CI, 2.673–14.217], *P* < 0.001).

**Table 2:** Univariable and multivariable logistic analyses of possible predictors for ICAS-O.

**Characteristics**	**Uni-variable**			**Multivariable**	
	**ICAS-O** **(*n* = 135)**	**Non-ICAS-O** **(*n* = 179)**	***P*-value**	**Adjusted OR** **(95%CI)**	***P*-value**
Age, mean (SD)	61.5 (10.9)	68.3 (13.5)	<0.001*	1.00 (0.976–1.032)	0.788
Gender female, *n* (%)	27 (20.0)	88 (49.2)	<0.001*	0.349 (0.155–0.787)	0.011*
Previous ischemic stroke, *n* (%)	31 (23.0)	31 (17.3)	0.224		
Hypertension, *n* (%)	94 (69.6)	93 (52.0)	0.001*	1.85 (0.933–3.675)	0.078
Diabetes, *n* (%)	37 (27.4)	26 (14.5)	0.004*	1.94 (0.840–4.479)	0.121
Atrial fibrillation, *n* (%)	4 (3.0)	104 (58.1)	<0.001*	0.01 (0.003–0.046)	<0.001*
Current smoking, *n* (%)	62 (45.9)	44 (24.6)	<0.001*	1.00 (0.463–2.147)	0.993
Baseline NIHSS, median (IQR)	9 (5, 16)	15 (11, 19)	<0.001*	0.93 (0.887–0.973)	0.002*
Posterior circulation, *n* (%)	41 (30.4)	12 (6.7)	<0.001*	4.04 (1.454–11.242)	0.007*
VBAC, *n* (%)	68 (50.4)	45 (25.1)	<0.001*	6.16 (2.673–14.217)	<0.001*

ICAS-O, intracranial arthrosclerosis related occlusion; SD, standard deviation; NIHSS, national institute of health stroke scale; IQR, interquartile range; VBAC, vertebrobasilar artery calcification; and OR, odds ratio. *p < 0.05 indicates statistical significance.

**Figure 2:**
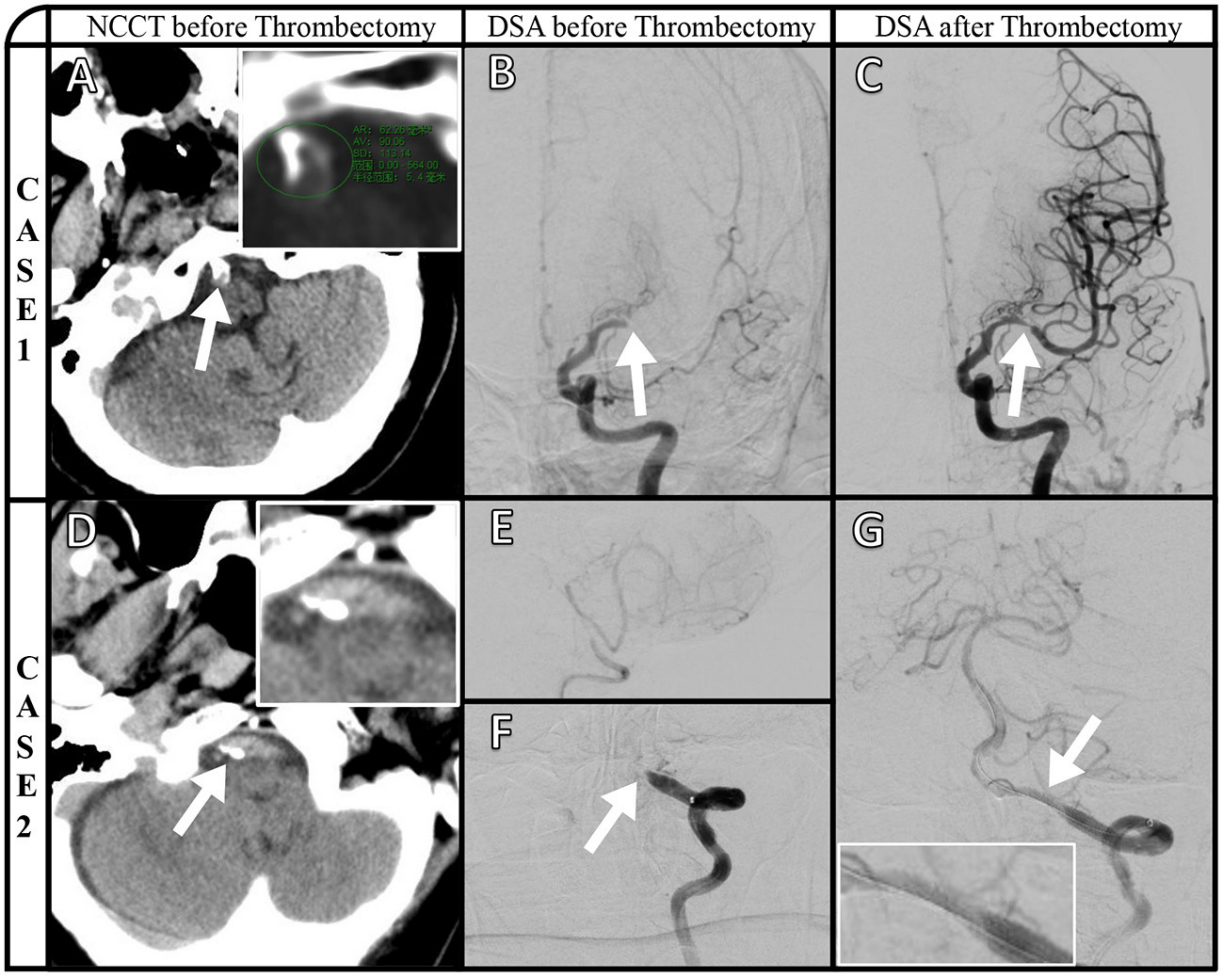
Representative images of VBAC and ICAS-O in thrombectomy (CASE1 and CASE2). Axial NCCT image **(A)** and magnified view (insert in A) show basilar artery calcification, and the highest CT value is 564HU. Posteroanterior left internal carotid artery angiography **(B)** manifested left middle cerebral artery occlusion. The stenosis **(C)** after thrombectomy demonstrates the etiology is ICAS occlusion. Axial NCCT imaging from a different patient **(D)** and magnified view (inset in D) show left vertebral artery calcification. Right vertebral artery angiography **(E)** shows that only supply posterior inferior cerebellar artery, and posteroanterior left artery angiography shows **(F)** V4 occlusion. The stent **(G)** and magnified view (insert in G) released *in situ* ICAS occlusion.

Representative images of VBAC and ICAS-O in thrombectomy (CASE1 and CASE2) are shown in Figure 2. Axial NCCT image (A) and magnified view (insert in A) show basilar artery calcification, and the highest CT value is 564HU. Posteroanterior left internal carotid artery angiography (B) manifested left middle cerebral artery occlusion. The stenosis (C) after thrombectomy demonstrates the etiology is ICAS occlusion. Axial NCCT imaging from a different patient (D) and magnified view (inset in D) show left vertebral artery calcification. Right vertebral artery angiography (E) shows that only supply posterior inferior cerebellar artery, and posteroanterior left artery angiography shows (F) V4 occlusion. The stent (G) and magnified view (insert in G) released *in situ* ICAS occlusion.

**Figure 3:**
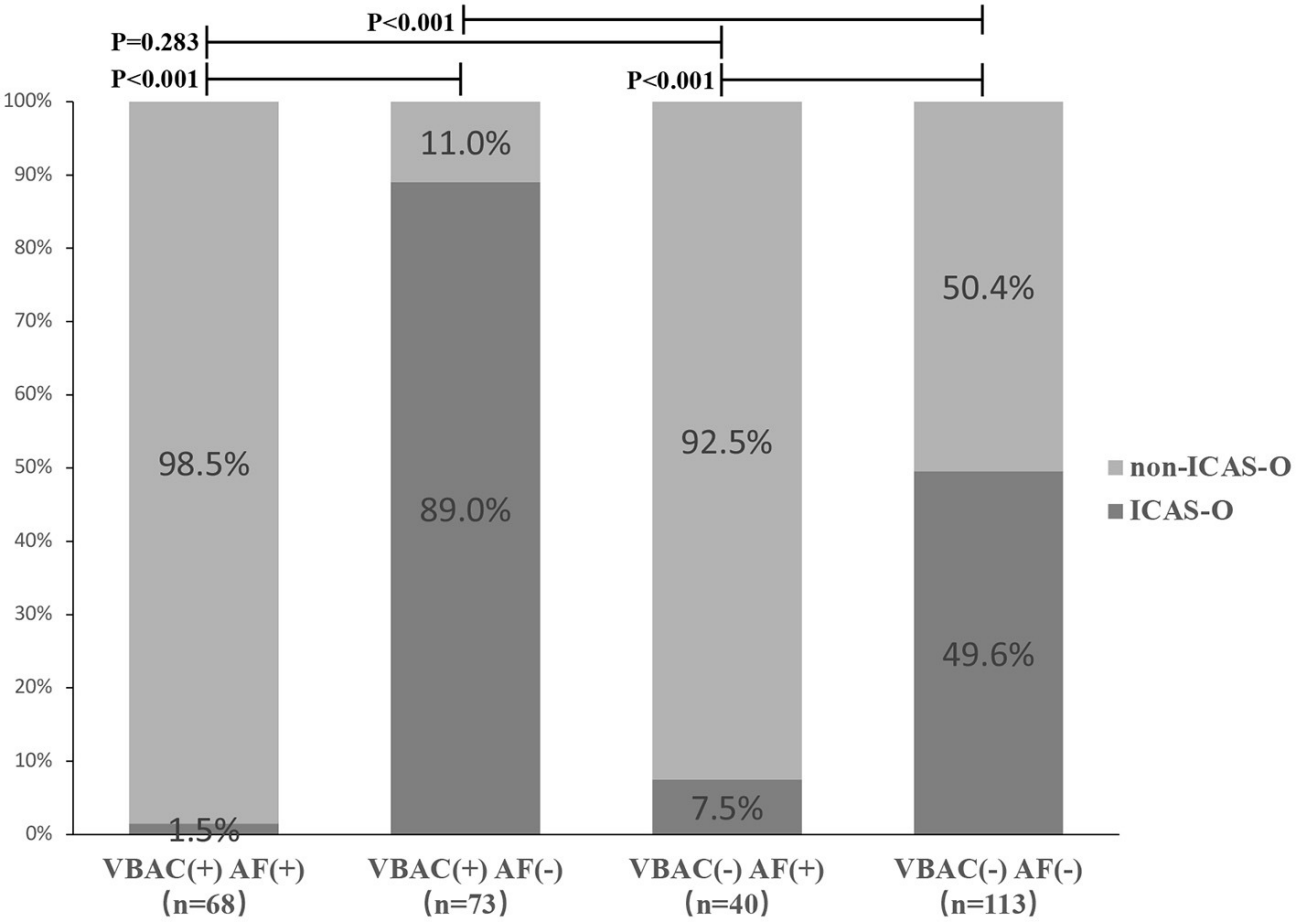
Proportions of ICAS-O classified according to with and without vertebrobasilar calcification (VBAC) and atrial fibrillation (AF). Non-ICAS-O more frequently occurred in VBAC(+) AF(+) group and VBAC(-) AF(+) group compared to the VBAC(+) AF(-) group (*P* < 0.001) and VBAC(-) AF(-) group (*P* < 0.001). The difference between the VBAC(+) AF(+) group and the VBAC(-) AF(+) group was not statistically significant (*P* = 0.283). However, the VBAC(+) AF(-) group shows ICAS-O more frequently than the VBAC(-) AF(-) group (*P* < 0.001).

Proportions of ICAS-O classified according to with and without VBAC and arterial fibrillation (AF) are shown in Figure 3. Non-ICAS-O more frequently occurred in VBAC(+) AF(+) group and VBAC(-) AF(+) group compared to the VBAC(+) AF(-) group (P <0.001) and VBAC(-) AF(-) group (*P* < 0.001). The difference between the VBAC(+) AF(+) group and the VBAC(-) AF(+) group was not statistically significant (*P* = 0.283). However, the VBAC(+) AF(-) group shows ICAS-O more frequently than the VBAC(-) AF(-) group (*P* < 0.001).

**Figure 4:**
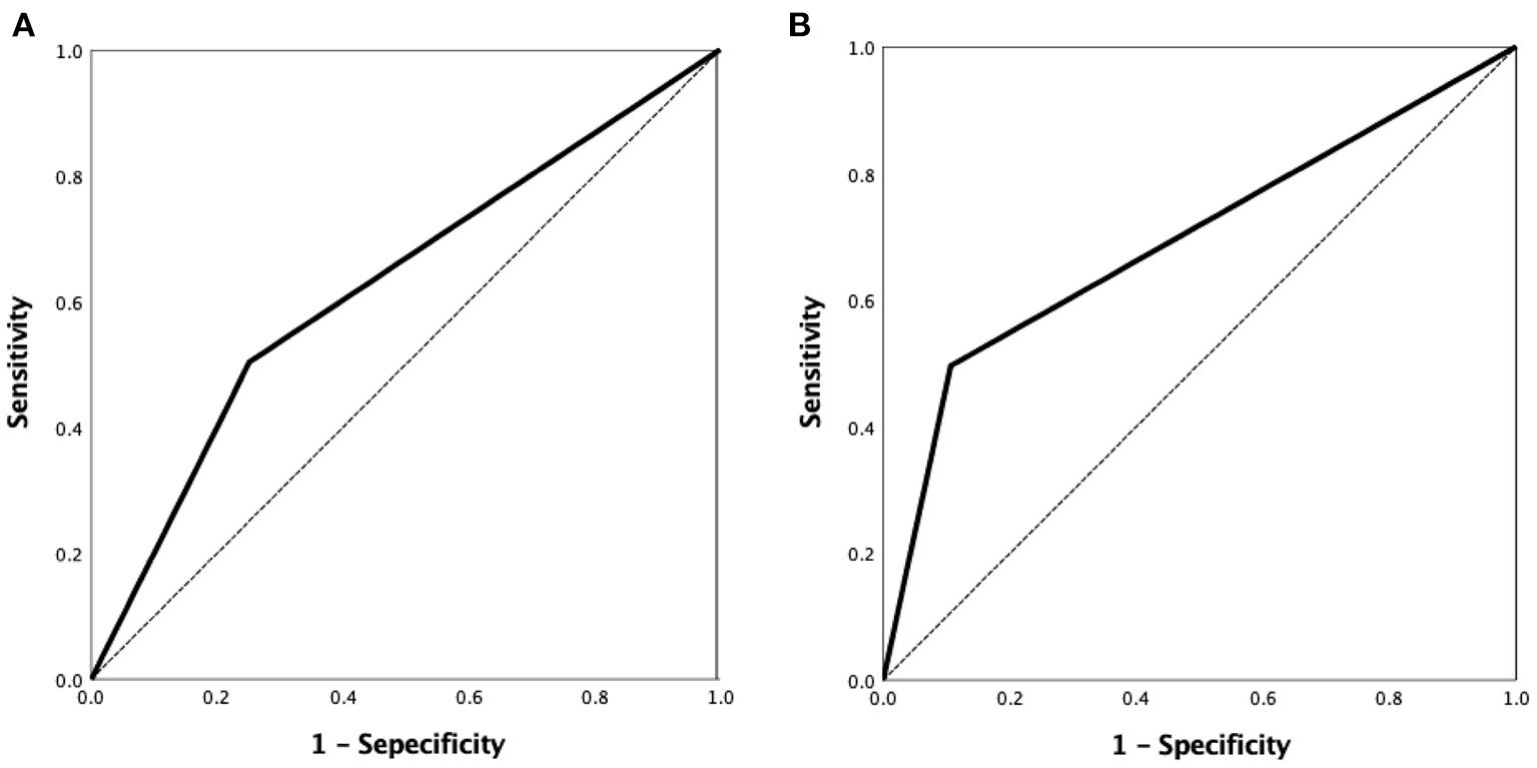
ROC curve analysis of the VBAC to predict ICAS-O was performed with the area under curve (AUC) of 0.626 (95% confidence interval [CI] 0.570–0.680, *p* < 0.001) **(A)** and 0.695 (95%CI 0.627–0.757) in the non-AF group **(B)**. ROC, receiver-operating characteristic.

A ROC curve analysis was used to evaluate the VBAC to predict the ICAS-O with an AUC of 0.626 (95% confidence interval [CI] 0.570–0.680, *p* < 0.001) (Figure 4A) and 0.695 (95%CI 0.627–0.757) in the non-AF subgroup (Figure 4B).

## Discussion

The major findings of the current study demonstrate that VBAC is associated with ICAS-O in patients undergoing endovascular therapy (EVT). Patients with VBAC had more than 6-fold higher odds of ICAS-O between patients with and without VBAC. Moreover, the probability of ICAS-O in patients with AF(-)VBAC(-) was 49.6% compared with 89.0% in AF(-)VBAC(+), suggesting that VBAC is an important clue for early detection of ICAS-O in AF(-) patients.

The probability of VBAC occurring in our study was 36%, which has been identified in 21.0% of elderly community-dwelling people (16), 39.1% of people presenting posterior circulation large vessel occlusion (18), and 54.6% in ischemic stroke patients (19). Factors affecting VBAC are age, hypertension, and diabetes, findings that are consistent with the study by Janine et al. (16). However, there was no significant difference in current smoking, which may be related to differences in the study's target population. Apart from precipitated calcium deposits in necrotic tissue, calcifications may have “bone-like” structures driven by hematopoietic cells (20), thereby appearing as high densities on CT scans along the artery. Furthermore, previous studies have suggested VBAC as associated with intracranial atherosclerosis (13, 14).

ICAS is a progressive disease that leads to arterial changes ranging from mild wall thickening to hemodynamically luminal stenosis (17, 21). LVO is typically related to in situ thrombotic occlusions, which occur when unstable plaques rupture in patients with advanced ICAS (22). ICAS-O is an important cause of LVO, especially in Asian populations where ICAS-O in the anterior circulation is expected to be between 17.6 and 33.6% (4, 23), 24.1–71.4% in the posterior circulation (8, 9, 24), and 43% in this study. In our data, we included 2 patients with M2 occlusion assessed on preoperative imaging, but M3 segment occlusion was confirmed after DSA. A retrospective systematic review and meta-analysis of 1967 patients (7) revealed that ICAS-O was associated with younger age, hypertension, diabetes, and smoking but negatively associated with AF. Our study showed a similar trend for ICAS-O related to the aforementioned risk factors. In another comparative study, there were more men in the ICAS-O group [2], and the baseline NIHSS score was significantly lower in the ICAS group than in the non-ICAS group (25). Lower NIHSS score in ICAS-O may partially be explained by preexisting collateral compensation in these patients. Patients with ICAS-O had better collateral flow than those without ICAS-O, presumably because ICAS requires a longer time for complete arterial occlusion, which allows for the development of adequate collateral flow before the onset of acute stroke (17). Furthermore, previous literature has reported that intracranial atherosclerotic disease is more closely associated with posterior circulation involvement (2).

Differentiating between ICAS-O and non-ICAS-O is essential for endovascular treatment strategies. Intraarterial therapy may include the use of different front-line thrombectomy techniques and potentially require rescue treatments (17). Alternatively, intracranial stenting can be performed without attempting mechanical thrombectomy. Notably, the primary stenting strategy was used in all cases of ICAS-O in a few studies (26, 27). The fundamental rationale for this was that the stent retriever could potentially lead to vessel injury on the ICAS lesion, resulting in intense reocclusion or dissection. Furthermore, these reports found that the primary stenting strategy was significantly associated with a shorter procedure time and a more favorable outcome than the primary stent retriever strategy.

ICAS-O treatment is technically more complex than embolism-related occlusion (EMB-O), and 36.9% of patients with ICAS-O will present reocclusion compared with 2.7% of patients with non-ICAS-O as identified by a meta-analysis (7). Thrombectomy using a stent retriever was successful in only 28.9% of patients with ICAS-O (4), and may potentially cause more procedural complications (28). Patients with ICAS-O may require rescue treatment, including emergency balloon angioplasty, emergency stenting, and arterial or intravenous use of GPI (8, 231, 9, 4, 10). Due to the major components of clots being formed by *in situ* thrombosis involving platelets and fibrin, intra-arterial infusion of an antiplatelet drug at the occlusion site may be a reasonable therapeutic option in patients with *in situ* thrombosis (29). Previous microscopy to high-resolution magnetic resonance imaging studies has shown histological evidence of disruption of the fibrous cap, intraplaque hemorrhage, and subendothelial stripping of the involved vessel segment in patients undergoing ICAS-O thrombectomy that may lead to early reocclusion (30). Therefore, early detection of ICAS-O and setting an optimal strategy appropriate for ICAS might be a key to faster recanalization, leading to better clinical outcomes in patients with ICAS-O. From the summary of our data, the presence of VBAC in preoperative NCCT without AF is more likely to have an etiology of ICAS-O.

Certain limitations of this study should be mentioned and considered in future developments. First, the Asian single-center retrospective design has the potential to introduce selection bias and limit the generalizability to other races. In future investigations, multi-racial prospective studies are needed to verify these results. Second, to ensure the detection rate of VBAC, a CT scan of 64 rows and above is required, and the scanning range should conclude the initial segment of V4 at the foramen magnum. Additionally, CT equipment may not meet the requirements in select primary stroke centers and partial preoperative CT did not conclude the foramen magnum that the V4 segment calcification was omitted. Third, this study did not include blood-related indicators such as cholesterol due to the influence of dietary factors. Furthermore, it is challenging to obtain accurate indicators during the emergency thrombectomy. We preliminarily determined the etiology of large vessel occlusion through medical history, electrocardiogram, and head CT, which may be more in line with the actual clinical workflow. Fourth, the patients which do not have recanalization (remain occluded), they may have ICAS-O but were excluded in this analysis. Fifth, we did not measure VBAC volume because calcification in the V4 segment of the vertebral artery was not fully included in many cases. The volume of the VBAC is important and has been described as being associated with worse outcomes (31). In further study, we intend to contain the V4 segment of the vertebral artery completely and measure the VBAC volume accurately.

## Conclusions

To our knowledge, this study is the first to demonstrate that VBAC is an independent risk factor for ICAS-O in patient underwent thrombectomy. According to our findings from patients eligible for endovascular treatment, we first determined whether the patient had AF. Patients with AF should be considered directly for EMB-O regardless of the combination of VBAC. Our findings identified that patients free of AF with VBAC present on cranial CT have an 89% probability of ICAS-O, especially in patients with posterior circulation. From these analyses, it is possible to prepare for subsequent surgical strategies such as the application of GPIs, balloons, stents. Furthermore, referral to a comprehensive stroke center should be considered if the armamentarium and skills of the primary stroke center are not beneficial in such specific patient cases.

## Data availability statement

The original contributions presented in the study are included in the article/supplementary material, further inquiries can be directed to the corresponding author/s.

## Ethics statement

The studies involving human participants were reviewed and approved by the local Institutional Review Board at the Affiliated Hospital of Northwest University (No. SYXSLL-2018-010). Written informed consent for participation was not required for this study in accordance with the national legislation and the institutional requirements.

## Author contributions

MC and YT: conception and design. NH: data collection and article draft. GZ, SY, HM, HG, XiaoZ, SL, YW, XF, YY, YG, WS, and XiaobZ: manuscript review and revision. All authors contributed to the article and approved the submitted version.

## Funding

This study was partially supported by grants from Xi'an Science and Technology Plan Project [21YXYJ0004].

## Conflict of interest

The authors declare that the research was conducted in the absence of any commercial or financial relationships that could be construed as a potential conflict of interest.

## Publisher's note

All claims expressed in this article are solely those of the authors and do not necessarily represent those of their affiliated organizations, or those of the publisher, the editors and the reviewers. Any product that may be evaluated in this article, or claim that may be made by its manufacturer, is not guaranteed or endorsed by the publisher.
